# Prognostic value of amplitude‐integrated EEG in neonates with high risk of neurological sequelae

**DOI:** 10.1002/acn3.50989

**Published:** 2020-02-07

**Authors:** Xiao Yuan, Wenqing Kang, Juan Song, Jing Guo, Lanlan Guo, Ruili Zhang, Shasha Liu, Yaodong Zhang, Dapeng Liu, Yong Wang, Xue Ding, Huimin Dong, Xi Chen, Yanchao Cheng, Xiaoli Zhang, Falin Xu, Changlian Zhu

**Affiliations:** ^1^ Henan Key Laboratory of Child Brain Injury Third Affiliated Hospital and Institute of Neuroscience Zhengzhou University Zhengzhou 450052 China; ^2^ Neonatal Intensive Care Unit, Zhengzhou Key Laboratory of Newborn Disease Research Children's Hospital Affiliated to Zhengzhou University Zhengzhou 450018 China; ^3^ Center for Brain Repair and Rehabilitation Institute of Neuroscience and Physiology Sahlgrenska Academy Gothenburg University Gothenburg 40530 Sweden; ^4^ Department of Women's and Children's Health Karolinska Institutet Stockholm 2995 Sweden

## Abstract

**Objective:**

To determine the efficacy and the prognostic value of amplitude‐integrated electroencephalography (aEEG) in term and near‐term neonates with high risk of neurological sequelae.

**Methods:**

Infants of ≥35 weeks of gestation diagnosed with neonatal encephalopathy or with high risk of brain injury were included. All eligible infants underwent aEEG within 6 h after clinical assessment. The infants were followed up 12 months to evaluate neurological development.

**Results:**

A total of 250 infants were eligible, of which 85 had normal aEEG, 81 had mildly abnormal aEEG, and 84 had severely abnormal aEEG. Of these infants, 168 were diagnosed with different neonatal encephalopathies, 27 with congenital or metabolic diseases, and 55 with high risk of brain injury. In all, 22 infants died, 19 were lost to follow‐up, and 209 completed the follow‐up at 12 months, of which 62 were diagnosed with a neurological disability. Statistical analysis showed that severely abnormal aEEG predicted adverse neurological outcome with a sensitivity of 70.2%, a specificity of 87.1%, a positive predictive value of 75.6%, and a negative predictive value of 83.7%.

**Interpretation:**

aEEG can predict adverse outcomes in high‐risk neonates and is a useful method for monitoring neonates with high risk of adverse neurological outcomes.

## Introduction

Neonatal encephalopathy (NE) is a clinical constellation of neurological dysfunctions in infants born at or beyond 35 weeks of gestation and that occur secondary to peripartum or intrapartum insult.^1^, ^2^ NE encompasses not only hypoxic‐ischemic encephalopathy (HIE)^3^ but also bilirubin encephalopathy (BE),^4^ intracranial hemorrhage (ICH), hypoglycemia encephalopathy,^5^ purulent meningitis,^6^ and epileptic encephalopathies.^7^ NE is often complicated with different congenital and metabolic diseases,^8^, ^9^ and it manifests as difficulty with initiating and maintaining respiration, altered mental status, changes in tone, depressed primitive reflexes, and seizures. NE has been estimated to cause 287,000 deaths and over 50 million cases of childhood disability around the world every year,^10^ and it is the second most common cause of avoidable long‐term neurological morbidity worldwide.^11^ Prognostication in these high‐risk populations remains rather full of uncertainties, despite the availability of an increasing number of laboratory evaluations and neuroimaging techniques.^12^

Amplitude‐integrated electroencephalography (aEEG) is a safe and generally well‐accepted neurophysiologic monitoring applied in preterm and term infants in neonatal intensive care units (NICUs), and the increasing numbers of newborns with associated risks of neurological disorders^13^ and the availability of new therapeutic strategies – such as trials of neuroprotection with hypothermia^14^ and antiepileptic drugs^15^ – suggest a growing need for aEEG. The interpretation of an aEEG tracing includes three categories – the classification of the background pattern, the identification of sleep–wake cycling (SWC), and the identification of seizures – all of which depend upon the infant's gestational age^16^, ^17^ and different pathophysiological conditions.^18^, ^19^, ^20^, ^21^, ^22^, ^23^ Previous studies reported that continuous extremely low voltage, flat tracing, or burst suppression of aEEG background patterns is correlated with unfavorable neurological outcomes in asphyxiated neonates,^24^, ^25^, ^26^ and recurrent seizures and status epilepticus have been shown to be indicators of poor neurodevelopmental outcomes,^27^ while fully developed SWC has been shown to be a good prognostic indicator.^28^ In this study, we incorporated the above three individual categories of an aEEG recording in order to objectively and comprehensively evaluate the cerebral function of newborns with other injuries than just HIE in order to determine the prognostic certainty of aEEG.

## Subjects and Methods

### Patient population

Term or near‐term infants of ≥35 weeks of gestational age and who were diagnosed with NE or who were at high risk of brain injury (such as severe hyperbilirubinemia, sustained hypoglycemia, severe sepsis, or neonatal seizures) but not diagnosed with NE were included from the NICUs of the Third Affiliated Hospital and Children's Hospital Affiliated of Zhengzhou University. The treatment protocols including hypothermia, exchange transfusion, antiepileptic drugs, and symptomatic support were in keeping with the criteria of expert consensus on the clinical diagnosis and treatment of corresponding clinical symptoms in China. The criteria for whole‐body hypothermia^29^ were (1) an Apgar score ≤3 at 1 min or ≤5 at 5 min or continued need for resuscitation, (2) an umbilical cord blood or arterial blood pH < 7.0 within 1 h after birth or a base deficit >16 mmol/L, and (3) assessed with moderate/severe HIE or abnormal aEEG. Whole‐body hypothermia was initiated within 12 h of age^30^ using a cooling mattress. A rectal temperature of 33°C–34°C was achieved within 60 min and maintained for 72 h, followed by rewarming at 0.5°C per hour until a rectal temperature of 36.5°C for more than 6 h. All eligible infants underwent aEEG and were tracked until 12 months for neurodevelopmental outcomes. The study was approved by the ethics committee of the Third Affiliated Hospital of Zhengzhou University.

### Medical records

The data from the medical records of the infants included in this study were categorized into baseline characteristics (such as gestational age at birth, birth weight, sex, and mode of delivery), perinatal antecedents, and high‐risk factors for brain injury. Perinatal antecedents included placental abruption, premature rupture of membrane, abnormal amniotic fluid, nuchal cord, fetal distress, and gestational complications. High‐risk factors for brain injury included perinatal asphyxia, severe hyperbilirubinemia, intracranial hemorrhage, sustained hypoglycemia, severe sepsis, neonatal seizures, and congenital or metabolic disorders. Asphyxia was determined by the combination of Apgar score and pH of blood gas analysis. Mild asphyxia was defined as an Apgar score ≤7 at 1 or 5 min and pH < 7.2 within 1 h after birth, and severe perinatal asphyxia was defined as an Apgar score ≤3 at 1 min or ≤5 at 5 min and pH < 7.0 or base deficit >16 mmol/L. Severe hyperbilirubinemia was defined as bilirubin concentrations at or near the exchange transfusion threshold based on postnatal age or any elevated total serum bilirubin associated with the early signs of mild acute bilirubin encephalopathy.^4^ Intracranial hemorrhage included neonates with subdural, subarachnoid, subependymal, intraventricular, or intraparenchymal hemorrhage.^31^, ^32^ Sustained hypoglycemia included neonates unable to consistently maintain a preprandial plasma glucose concentration >50 mg/dL up to 48 h of age and >60 mg/dL after 48 h of age or those in need of intravenous dextrose therapy.^5^ Severe sepsis was defined as sepsis with cardiovascular organ dysfunction or acute respiratory distress syndrome or two or more other organ dysfunctions.^33^ Neonatal seizures were those with clinical seizures and confirmed by electrographic seizures on aEEG.

### Amplitude‐integrated electroencephalography

The aEEG and the raw EEG trace was acquired by manual application of individual electrodes according to the 10–20 system modified for neonates, and recorded for at least 4 h with a NicoletOne device (Nicolet Biomedical Inc., Madison, WI, US) within 6 h after clinical diagnosis or assessment of neurological injury. We graded the aEEG tracing according to amplitude, electrographic seizure, and sleep–wake cycling (SWC). Normal amplitude was those with upper margin >10 *μ*V and lower margin >5 *μ*V. SWC is characterized by smooth sinusoidal variations, mainly in the minimum amplitude. The broader bandwidth represents quiet sleep, and the narrower bandwidth active sleep. A cycle duration of more than 20 min of clearly identifiable sinusoidal variations between quiet sleep and active sleep represents developed SWC. Electrographic seizure was defined as an abrupt rise in the minimum amplitude with or without a simultaneous rise in the maximum amplitude followed by a short period of decreased amplitude in the aEEG and simultaneously repetitive spikes or sharp waves or activity with duration of at least 5–10 sec in the raw EEG.^34^

Normal aEEG was interpreted as normal amplitude, developed SWC, and no electrographic seizures; mildly abnormal was interpreted as mildly abnormal amplitude (upper margin >10 *μ*V and lower margin <5 *μ*V), immature SWC (some, but not fully developed as compared to normative gestational age representative data), or normal amplitude with electrographic seizures; and severely abnormal aEEG was interpreted as severely abnormal amplitude (upper margin <10 *μ*V and lower margin <5 *μ*V) without SWC, including burst suppression (discontinuous activity with lower margin at a constant 0–1 *μ*V and a burst amplitude >25 *μ*V), flat trace (electrical silence), continuous low voltage (continuous very low amplitude activity at about 5 *μ*V or below 5 *μ*V), status epilepticus (continuously ongoing seizure activity >30 min), or mildly abnormal amplitude with electrographic seizures.^35^

### Follow‐up

All surviving infants were tracked every 3 months for assessment of their physical, behavioral, and neurological development. Bayley Scales of Infant Development (2nd edition^36^) were used to evaluate the motor functions and mental developmental index (MDI) at 12 months of age. Those with a psychomotor developmental index <70 were deemed to have psychomotor retardation and to need further visits with rehabilitation physicians for subsequent diagnosis and classification of cerebral palsy (CP).^37^ Hearing ability was based on parental reports and auditory brainstem response measurements. Deafness was defined as a hearing disability that required amplification. Blindness was defined as a corrected visual acuity less than 0.05. Adverse neurological outcome was defined as death due to damage to the central nervous system or survival with at least one of the following complications: CP, epilepsy, auditory disorder, visual anomaly, MDI < 70, or psychomotor retardation.

### Statistical analysis

Statistical analysis was performed with SPSS 21.0 software. Quantitative data with normal distribution were presented as means ± SD and analyzed for statistical significance using an independent‐samples *t* test, while non‐normally distributed variables were presented as medians (quartile range) and were compared by Mann–Whitney *U*‐test. For categorical variables, we made statistical comparisons using the chi‐squared test. Logistic regression models were constructed to evaluate the associations between severe aEEG abnormality and adverse neurological outcomes. The receiver‐operator characteristic (ROC) curve was calculated to evaluate the predictive value of severely abnormal aEEG. All statistical tests were two‐sided, and *P* < 0.05 was considered statistically significant.

## Results

### General characteristics

**Figure 1:**
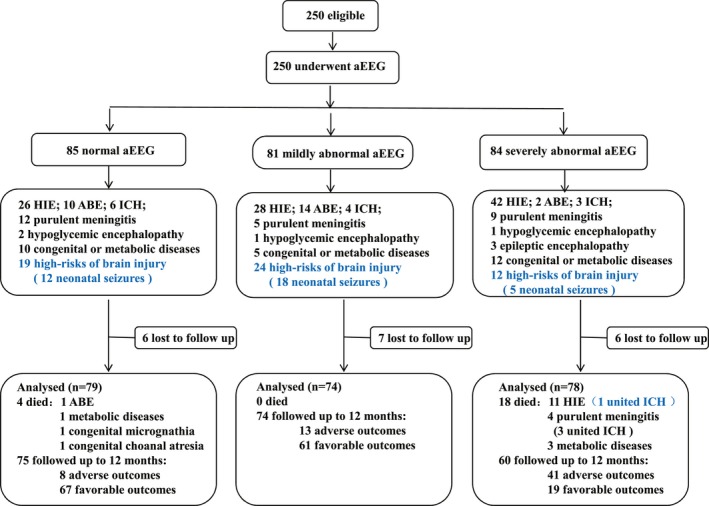
Study flow. The schematic flowchart depicting the numbers of infants who underwent aEEG divided into three categories according to severity of aEEG and followed up to 12 months. aEEG, amplitude‐integrated electroencephalography; HIE, hypoxic‐ischemic encephalopathy; ABE: acute bilirubin encephalopathy; ICH, intracranial hemorrhage; PVL: periventricular leukomalacia.

A total of 250 infants at high risk of neurological sequelae underwent aEEG and were enrolled during the study period, with a median (quartile range) gestational age of 39.3 (1.9) weeks, a median (quartile range) birth weight of 3300 (562) g, and a median (quartile range) postnatal age at admission of 5.0 (8.1) days. There were 168 infants diagnosed with different types of NE (including 96 HIE, 26 BE, 13 ICH, 4 hypoglycemia encephalopathy, 26 purulent meningitis, and 3 epileptic encephalopathies), 27 were diagnosed with congenital or metabolic diseases, and 55 were at high risk of brain injury predominantly due to neonatal seizures. Adverse neurological outcome had a significant association with gestational age, birth weight, and congenital or metabolic diseases (*P* < 0.05) (Table 1). On visual analysis of the aEEG, 85 infants were normal, 81 were mildly abnormal, and 84 were severely abnormal. In all, 22 infants died of brain injury during the study period, and 209 completed the follow‐up at 12 months, with 29.7% showing some form of neurological disability (survival with at least one of the following complications: CP, epilepsy, auditory disorder, visual anomaly, MDI < 70, or psychomotor retardation) (Fig. 1).

**Table 1:** Baseline characteristics.

Variables	Eligible infants (*n* = 250)	Neurological outcomes (*n* = 231)		
		Adverse (*n* = 84)	Favorable (*n* = 147) 147)	*P* value
Gestational age (weeks)	39.3 (1.9)*	39.1 (2.0)*	39.6 (1.1)*	0.031
Birth weight (g)	3300 (562)*	3175 (700)*	3300 (550)*	0.044
Male *n* (%)	155 (62.0%)	55 (65.5%)	91 (61.9%)	0.588
Caesarean section *n* (%) delivery	118 (47.2%)	44 (52.4%)	61 (41.5%)	0.110
Age at admission (days)	5.0 (8.1)*	4.0 (12.0)*	5.0 (9.0)*	0.397
Blood glucose at admission (mmol/L)	3.9 ± 1.2 (0.2–9.8)	3.7 ± 1.2	4.0 ± 1.2	0.056
Placental abruption *n* (%)	7 (2.8%)	3 (3.6%)	3 (2.0%)	0.784
Prolonged rupture of membrane *n* (%)	39 (15.6%)	10 (11.9%)	26 (17.7%)	0.244
Abnormal amniotic fluid *n* (%)	96 (38.4%)	37 (44.0%)	52 (35.4%)	0.193
Nuchal cord *n* (%)	26 (10.4%)	10 (11.9%)	14 (9.5%)	0.568
Gestational complications *n* (%)	17 (6.8%)	6 (7.1%)	10 (6.8%)	0.922
Perinatal asphyxia *n*. (%)	106 (42.4%）	40 (47.6%)	60 (40.8%)	0.315
Severe hyperbilirubinemia *n* (%)	26 (10.4%）	9 (10.7%)	17 (11.6%)	0.844
Intracranial hemorrhage *n* (%)	13 (5.2%）	2 (2.4%)	11 (7.5%)	0.186
Severe sepsis *n* (%)	32 (12.8%）	7 (8.3%）	21 (14.3%）	0.182
Sustained hypoglycemia *n* (%)	7 (2.8%）	1 (1.2%)	3 (2.0%)	1.000
Neonatal seizures *n* (%)	38 (15.2%）	8 (9.5%)	25 (17.0%)	0.118
Congenital or metabolic diseases *n* (%)	27 (10.8%）	17 (20.2%)	9 (6.1%)	0.001
Severely abnormal aEEG *n* (%)	84 (33.6%)	59 (70.2%)	19 (12.9%)	0.000

Adverse neurological outcome is defined as death due to injury to the central nervous system or survival with one or more of CP, epilepsy, auditory disorder, visual anomaly, MDI < 70, or psychomotor retardation. Gestational complications were those diagnosed with pregnancy‐induced hypertension, gestational diabetes mellitus, and/or inflammatory disease. Quantitative data with normal distribution are presented as means ± SD.

* Quantitative data with non‐normal distribution are presented as medians (quartile range). Probability value is for adverse neurological outcome versus favorable neurological outcome using Student's *t* test/Mann–Whitney *U*‐test or chi‐square test. *P* < 0.05 was considered statistically significant.

### Determination of aEEG

Among 81 cases with mildly abnormal aEEG, mildly abnormal amplitude was found in 14 cases, immature SWC was found in 47 cases, and normal amplitude with electrographic seizures was found in 20 cases. Among the 84 cases with severely abnormal aEEG, 65 infants had severely abnormal amplitude without SWC, 9 had burst suppression, 5 had status epilepticus, 3 had continuous low voltage, and 2 had mildly abnormal amplitude with electrographic seizures.

### Neurological outcomes of severe aEEG abnormality

Regarding the outcomes, death occurred in 18 of the 78 infants (23.1%) with severely abnormal aEEG. Further analysis was done for the 209 surviving infants who completed the follow‐up at 12 months of age, including 149 cases without severely abnormal aEEG, and 60 cases with severely abnormal aEEG. The incidence rate of CP, epilepsy, psychomotor retardation, and intellectual retardation all increased as the severity of aEEG increased (*P* < 0.001). The incidences of auditory disorders were similar regardless of the severity of aEEG (*P* = 0.156) (Table 2). In all, 41 of 60 (83.3%) of the infants with severely abnormal aEEG had neurological disability (survival with at least one of the following complications: CP, epilepsy, auditory disorder, visual anomaly, MDI < 70, or psychomotor retardation at 12 months of age), while 14.1% (21 of 149) of infants with no severely abnormal aEEG had neurological disability. The severity of aEEG was significantly associated with both mortality and long‐term disability.

**Table 2:** Neurological outcomes according to aEEG severity.

Outcome	aEEG		*P* value
	Non‐severe abnormality (*n* = 166)	Severe abnormality (*n* = 84)	
Death, *n* (%)	4/153 (2.6%)	18/78 (23.1%)	<0.001
CP, *n* (%)	12/149 (8.1%)	23/60 (38.3%)	<0.001
Epilepsy, *n* (%)	3/149 (2.0%)	7/60 (11.7%)	0.013
Auditory disorder, *n* (%)	6/149 (4.0%)	6/60 (10.0%)	0.364
Visual anomaly, *n* (%)	0	11/60 (18.3%)	<0.001
Psychomotor retardation, *n* (%)	13/149 (8.7%)	27/60 (45.0%)	<0.001
MDI < 70, *n* (%)	11/149 (7.4%)	20/60 (33.3%)	<0.001
Adverse neurological outcome, *n* (%)	25/153 (16.3%)	59/78 (75.6%)	<0.001

CP, cerebral palsy; non‐severely abnormal aEEG, normal or mildly abnormal aEEG; probability value is for different severity of aEEG with neurological outcome using the chi‐square test. *P* < 0.05 was considered statistically significant. Adverse neurological outcome was defined as death due to damage to the central nervous system or survival with at least one of the following complications: CP, epilepsy, auditory disorder, visual anomaly, MDI < 70, or psychomotor retardation.

### Predictive ability of severely abnormal aEEG for adverse neurological outcome

Univariate logistic regression analysis showed that severely abnormal aEEG was a significant risk factor for death (adjusted regression coefficient: 2.414; S.E.: 0.574; *P* < 0.001; OR: 11.175; 95% CI [3.631–34.390]), CP (adjusted regression coefficient: 1.592; SE: 0.390; *P* < 0.001; OR: 4.914; 95% CI [2.288–10.553]), epilepsy (adjusted regression coefficient: 1.595; SE: 0.705; *P* < 0.001; OR: 4.930; 95% CI [1.238–19.627]), psychomotor retardation (adjusted regression coefficient: 1.741; SE: 0.375; *P* < 0.001; OR: 5.701; 95% CI [2.733–11.893]), and MDI < 70 (adjusted regression coefficient: 1.493; SE: 0.406; *P* < 0.001; OR: 4.451; 95% CI [2.007–9.873]), but not for auditory disorders or visual anomalies (*P* > 0.05) (Table 3). ROC analysis revealed that severely abnormal aEEG predicted adverse neurological outcome with an area under the ROC curve (AUC) of 0.787 and a Youden Index of 0.57 implying a sensitivity of 70.2%, a specificity of 87.1%, a positive predictive value of 75.6%, and a negative predictive value of 83.7% (Table 4).

**Table 3:** Univariate logistic regression analysis of severely abnormal aEEG for different outcomes.

Outcome	B	SE	*P*	OR	95% CI
Death	2.414	0.574	<0.001	11.175	3.631–34.390
CP	1.592	0.390	<0.001	4.914	2.288–10.553
Epilepsy	1.595	0.705	0.024	4.930	1.238–19.627
Auditory disorder	0.714	0.595	0.230	2.042	0.636–6.553
Visual anomaly	19.396	\	0.995	\	\
Psychomotor retardation	1.741	0.375	<0.001	5.701	2.733–11.893
MDI < 70	1.493	0.406	<0.001	4.451	2.007–9.873
Adverse neurological outcome	2.766	0.343	<0.001	15.899	8.123–31.118

B, adjusted regression coefficient; CI, confidence interval; OR, odds ratio; SE, standard error.

**Table 4:** Prediction of severely abnormal aEEG.

Outcome	AUC	Youden Index	Sensitivity	Specificity	PPV	NPV	Accuracy
Death	0.766	0.53	81.8%	71.3%	23.1%	97.4%	72.3%
CP	0.688	0.38	65.7%	71.9%	29.5%	92.2%	71.0%
Epilepsy	0.689	0.38	70.0%	67.9%	9.0%	98.0%	68.0%
Psychomotor retardation	0.704	0.41	67.5%	73.3%	34.6%	91.5%	72.3%
MDI < 70	0.678	0.36	64.5%	71.0%	25.6%	92.8%	70.1%
Adverse neurological outcome	0.787	0.57	70.2%	87.1%	75.6%	83.7%	81.0%

AUC, the areas under the ROC curve; NPV, negative predictive value; PPV, positive predictive value.

## Discussion

In this study, we analyzed the early predictive value of aEEG for term or near‐term newborn infants with encephalopathy or at high risk of brain injury. Our results demonstrate that aEEG is associated with both short‐term (neonatal death) and long‐term (at 12 months of age) adverse neurological outcomes and that it could serve as a routine bedside monitor for neonates with high risk of neurological sequelae.

It has been shown that hypothermia after asphyxia decreases mortality and improves survival without neurological disability^38^ and as a result lowers the prognostic value of early aEEG initiated before hypothermia.^14^, ^39^ Consistent with this, we also found that the prognostic value of early aEEG in infants before hypothermia was inferior to aEEG after hypothermia, with a sensitivity of 73.7% and a specificity of 71.9%. Furthermore, this study required that hypothermia starts within 12 h after asphyxia thus taking into consideration many factors that might delay the initiation of hypothermia, particularly for those living in rural areas where hypothermia devices may not be readily available. One might question the curative benefits of the delayed cooling time window, even though our previous study showed an apparently beneficial effect of hypothermia even when extended to 10 h of age.^40^ However, among our 250 infants, only 53 received hypothermia, so the possibility of hypothermia‐induced effects on our overall results was not significant. Lastly, the end point of neurological outcomes of 12 months was reassuring because the sensitivity of the 1‐year evaluation of the Bayley Scales of Infant Development in predicting the longer neurologic outcome was 96%.^41^ In conclusion, the predictive value of aEEG on adverse neurological outcomes was significant.

We found that the number of infants with auditory disorders at the 12‐month follow‐up showed no difference regardless of whether the aEEG tracing was severely abnormal or not because almost half of these disabled babies (5 of 12) were diagnosed with BE in the current study, and bilirubin toxicity to the auditory system was shown not to be correlated with cerebral cortical electrical activity.^42^ Although we found visual anomalies only in those with severely abnormal aEEG, we could not show that severe aEEG abnormality increased the risks of visual anomalies. In contrast, CP, epilepsy, and retarded mental or psychomotor developmental were all significantly more likely in infants with severely abnormal aEEG. This suggests that severely abnormal aEEG is related to unfavorable lifelong neurological sequelae. In addition, we found that the incidence of death in the neonatal period in the severely abnormal aEEG group (18/78, 23.1%) was significantly higher than in non‐severely abnormal aEEG group (4/153, 2.6%), which confirms that the severity of aEEG is associated with mortality.^43^ In short, early aEEG could predict not only short‐term outcomes but also long‐term outcomes in high‐risk infants.^44^, ^45^

Of the 59 severely abnormal aEEG infants among the 84 infants in the adverse neurological outcome group, 43 (43/59 = 72.9%) infants had severely abnormal amplitude without SWC. In terms of death, CP, epilepsy, and retarded mental or psychomotor developmental, we also found an absence of SWC on aEEG recordings in the majority of infants. Our findings agree with a previous study showing that most infants who lacked normal SWC either died or survived with significant neurological complications.^46^ Although we did not find any associations between aEEG and different neurological handicaps, we postulate that anomalous α, β, δ, or θ waves at different cortical regions in the raw EEG might give clinicians more information about different kinds of neurological handicaps or etiologies of NE, and this method might, therefore, to be of value for future studies.

Our previous study showed that the earlier the gestational age or the lower the body weight at birth, the greater the chances of suffering from neurological disability.^47^ Although we also observed that the incidence of adverse neurological outcome was associated with gestational age and birth weight in the current study, the differences in the adverse neurological outcome group compared to the favorable outcome group were not significant, which might be due to our inclusion criterion of gestational age ≥35 weeks. Additionally, our study also confirmed previous observations that the likelihood of suffering adverse neurological outcomes increases in those born with congenital or metabolic diseases.^48^

The primary limitation of this work may be that the timing of aEEG took little consideration that different therapeutic strategies, for example, hypothermia, might affect the efficacy of the prognostic value of aEEG. Another limitation might be our imbalanced proportion of infants suffering from HIE (96, 38.4%), which might be the reason that our current results are inconsistent with our previous study showing that undesirable antepartum or intrapartum events are risk factors for adverse neurological deficits.^49^ Moreover, we were unable to complete the analysis of the longer follow‐up, which might have given our current study more credibility in terms of the long‐term prognostic value of early aEEG. Lastly, our inability to use conventional video EEG to confirm the neonatal seizures might have increased the number of such diagnoses, which, in turn, would decrease the predictive value of aEEG. Consequently, we would likely need a larger sample size with a balanced etiology of NE, and an extended follow‐up in order to draw firmer conclusions. In spite of the above‐mentioned limitations, the current study has its strengths. Most important of all is that we deliberately chose a broad definition of NE that included infants with non‐asphyxia causes for their encephalopathy, and we showed that aEEG correlated with neurologic outcomes after NE from different causes. In addition, we stratified the aEEG assessment according to its background pattern, SWC, and the presence of electrographic seizures, which gave more objective and systemic results.

Taken altogether, this study provides evidence that aEEG is predictive of the short‐term and long‐term outcomes in high‐risk infants. We speculate, therefore, that aEEG might be a useful prognostic monitoring device for this population at high risk of neurological sequelae. Future research on the prognostic significance of aEEG will focus on overcoming the influence of different therapeutic strategies on aEEG and on prolonging the subsequent follow‐up to school age.

## Conflict of Interest

None of the authors have potential conflicts of interest to be disclosed.

## Authors' Contributions

CZ and XY designed the research; XY, WK, JS, JG, LG, RZ, SL, YZ, DL, YW, XD, HD, IC, YC, XZ, and FX were involved in data collection; XY and CZ analyzed the data; XY, JS, and CZ wrote the paper.
